# Generation of A Triple Insert Live Avian Herpesvirus Vectored Vaccine Using CRISPR/Cas9-Based Gene Editing

**DOI:** 10.3390/vaccines8010097

**Published:** 2020-02-21

**Authors:** Na Tang, Yaoyao Zhang, Yashar Sadigh, Katy Moffat, Zhiqiang Shen, Venugopal Nair, Yongxiu Yao

**Affiliations:** 1 Viral Oncogenesis Group & UK-China Centre of Excellence for Research on Avian Diseases, The Pirbright Institute, Pirbright, Surrey GU24 ONF, UK; tangna0543@163.com (N.T.); yaoyao.zhang@pirbright.ac.uk (Y.Z.); yashar.sadigh@pirbright.ac.uk (Y.S.); Kathryn.Moffat@pirbright.ac.uk (K.M.); 2 Postdoctoral workstation & UK-China Centre of Excellence for Research on Avian Diseases, Shandong Binzhou Animal Science and Veterinary Medicine Academy, Binzhou 256600, China; bzshenzq@163.com; 3 School of Animal Science and Technology, Guangxi University, Nanning 510642, China; 4 The Jenner Institute Laboratories, University of Oxford, Oxford OX3 7DQ, UK; 5 Department of Zoology, University of Oxford, Oxford OX1 3SZ, UK

**Keywords:** triple insert, HVT vectored vaccine, multivalent, CRISPR/Cas9

## Abstract

Herpesvirus of turkeys (HVT), used originally as a vaccine against Marek’s disease (MD), has recently been shown to be a highly effective viral vector for generation of recombinant vaccines that deliver protective antigens of other avian pathogens. Until the recent launch of commercial HVT-vectored dual insert vaccines, most of the HVT-vectored vaccines in the market carry a single foreign gene and are usually developed with slow and less efficient conventional recombination methods. There is immense value in developing multivalent HVT-vectored vaccines capable of inducing simultaneous protection against multiple avian pathogens, particularly to overcome the interference between individual recombinant HVT vaccines. Here we demonstrate the use of a previously developed CRISPR/Cas9 gene editing protocol for the insertion of ILTV gD-gI and the H9N2 AIV hemagglutinin expression cassettes into the distinct locations of the recombinant HVT-IBDV VP2 viral genome, to generate the triple insert HVT-VP2-gDgI-HA recombinant vaccine. The insertion, protein expression, and stability of each insert were then evaluated by PCR, immunostaining and Western blot analyses. The successful generation of the first triple insert recombinant HVT vaccine with the potential for the simultaneous protection against three major avian viral diseases in addition to MD is a major innovation in vaccination-based control of major poultry diseases.

## 1. Introduction

Poultry is one of the fastest growing sectors of livestock farming contributing hugely to the Global Food Security and alleviation of poverty. Poultry production is expected to grow by 24% in the next decade and it is estimated that poultry will account for the largest proportion of livestock output. This growth will only be feasible if losses from infectious diseases are successfully controlled, mainly through the use of effective vaccines. Conventional modified live and killed vaccines have been successful in preventing losses from most poultry diseases, however, they have many limitations including their inability to prevent horizontal and vertical transmission, increasing virulence and diversity of avian pathogens. Since the development of recombinant DNA technology, in the early 1980s, it was clear that recombinant vaccines could become an important new tool in protecting poultry against infectious diseases with increased efficiency [1]. The three live replicating viral vectors commonly used in poultry vaccination are HVT (herpesvirus of turkeys), FPV (fowlpox virus), and NDV (Newcastle disease virus), and the vectored vaccines are used to protect against diseases such as ND (Newcastle disease), IBD (infectious bursal disease), ILT (infectious laryngotracheitis), AI (avian influenza), and *Mycoplasma gallisepticum* infections. Among these, HVT is the most widely used vector due to its suitability and safety for both in ovo and subcutaneous hatchery administration, and it is effective in the presence of maternal antibodies and capable of providing life-long immunity. Recombinant HVT vaccines are generated either by conventional homologous recombination in virus-infected cells or through recombineering techniques using overlapping cosmid clones or on full-length genomes cloned as bacterial artificial chromosome (BAC) clones [2,3] which are often time-consuming and inefficient. Most of the recombinant HVT vaccines currently in use express single or double inserts of the foreign gene(s) [4], thus inducing protection against one or two pathogens in addition to the protection against Marek’s disease (MD) induced by the HVT vector. While HVT vector-based vaccines are efficient and are increasingly used by industry, the interference between the HVT vector backbones seriously limits the simultaneous use of sets of HVT vectored vaccine expressing antigens against multiple diseases [5]. Hence, there is a need for innovative and more efficient technologies for the rapid generation of multivalent vaccines to protect against a multitude of pathogens and diverse strains prevalent in different geographical regions of the world. 

Recent advances in gene editing technologies using CRISPR/Cas9 tools have expanded the possibilities of editing the DNA viral genomes. CRISPR/Cas9 has been used to manipulate genomes of several large DNA viruses including herpes simplex virus type I, adenovirus, pseudorabies virus, vaccinia virus, Epstein-Barr virus, guinea pig cytomegalovirus, Marek’s disease virus, Kaposi’s sarcoma-associated herpesvirus, duck enteritis virus and Infectious laryngotracheitis virus (ILTV) [6,7,8,9,10,11,12,13,14,15,16,17,18,19,20,21,22]. Recently, we have developed a rapid and efficient CRISPR/Cas9-mediated genome editing strategy for generating recombinant HVT [23,24,25]. Here we extend the application to generate triple gene insertion HVT vectored virus. We inserted glycoprotein D-glycoprotein I (gDgI) of ILTV and H9HA of H9N2 avian influenza virus, two protective antigens of important poultry pathogens being successfully inserted into HVT as vectored vaccines previously [4,26,27], sequentially into the previously constructed recombinant HVT-VP2, generating recombinant HVT-VP2-gDgI-HA with triple inserts. The expression and stability of the three inserts in the triple recombinant virus were evaluated by PCR, immunostaining and Western blot. This is the first report of the successful generation of a triple gene insert HVT recombinant and it offers the prospect of the rapid development of multivalent recombinant vaccines for the simultaneous protection of poultry against multiple avian viral pathogens. 

## 2. Materials and Methods 

### 2.1. Viruses and Cell Culture

HVT Fc126 strain was obtained from the Avian Disease and Oncology Laboratory (ADOL) East Lansing, MI, USA. The recombinant virus HVT-VP2 was constructed previously [23]. Primary chick embryo fibroblasts (CEF) were prepared from 10-day old embryos and maintained in M199 medium (Thermo Fisher Scientific, Waltham, MA, USA) supplemented with 5% fetal bovine serum (FBS, Sigma-Aldrich, Darmstadt, Germany), 100 units/ mL of penicillin and streptomycin (Thermo Fisher Scientific), 0.25 µg/mL Fungizone (Sigma), and 10% tryptose phosphate broth (Sigma).

### 2.2. Construction of sgRNAs and Donor Plasmids 

The gRNA targeting UL45/46 region, sg-A and donor plasmid pGEM-sgA-LoxP-RFP-VP2 were described previously [23]. The gRNA targeting the HVT65/66 and US2 regions of the HVT genome was designed using CRISPR guide RNA designing software (http://crispr.mit.edu/) and cloned into the CRISPR/Cas9 vector pX459-v2 by introducing synthesized oligo-DNA primers corresponding to the target sequence into *Bbs*I restriction sites [28]. The scramble sequences sg-B and sg-C which have no homology to human, chicken, pig genome, prokaryotic DNA sequence and viruses were cloned into px459-v2 in the same way. 

The donor plasmid pGEM-sgB-LoxN-GFP-HA containing green fluorescent reporter gene and AIV hemagglutinin gene expression cassettes was constructed in several steps. First, the oligo pairs sgB-LoxN-F and sgB-LoxN-R (containing the element of sgB+LoxN+PacI+LoxN+SfiI+spacer+SfiI+sgB) were annealed and cloned into pGEM-T-easy vector generating pGEM-sgB-LoxN vector. Then, the GFP expression cassette, released from pEF-GFP with restriction enzyme *Pac*I, was cloned into pGEM-sgB-LoxN via the *Pac*I site to generate pGEM-sgB-LoxN-GFP. An intermediate HA expression cassette was then constructed with the same promoter and poly A signal sequence for VP2 expression [23]. We first cloned two SfiI sites into pcDNA3.1(+) via NdeI and PmeI by annealing the oligo pairs SfiIx2-F and SfiIx2-R generating pcDNA3.1(+)-SfiIx2. The VP2 expression cassette was released from pGEM-sgA-LoxP-RFP-VP2 and cloned into pcDNA3.1(+)-SfiIx2 generating pcDNA3.1(+)-VP2. The VP2 coding sequence was then replaced with HA (from H9N2) coding sequence amplified with primer pairs HA-F and HA-R (Table 2) via NotI site generating pcDNA3.1-HA. Finally, the HA expression cassette was then transferred into pGEM-sgB-LoxN-GFP via SfiI generating pGEM-sgB-LoxN-GFP-HA. The procedure described above was followed for construction of the donor plasmid pGEM-sgC-Lox2272-GFP-gDgI containing GFP and ILTV gDgI expression cassettes. pGEM-sgC-Lox2272 was first constructed by cloning annealed oligo pairs sgC-Lox2272-F and sgC-Lox2272-R (containing the element of sgC+Lox2272+PacI+Lox2272+SfiI+spacer+SfiI+sgC). The GFP expression cassette was then cloned via the PacI site generating pGEM-sgC-Lox2272-GFP. Finally, synthesized gDgI expression cassette with SfiI sites were cloned generating pGEM-sgC-Lox2272-GFP-gDgI. The primer sequences used for guide RNA cloning and donor plasmid construction are listed in Table 1 and Table 2.

### 2.3. Generation of Recombinant HVT-VP2-gDgI-HA

**Figure 1:**
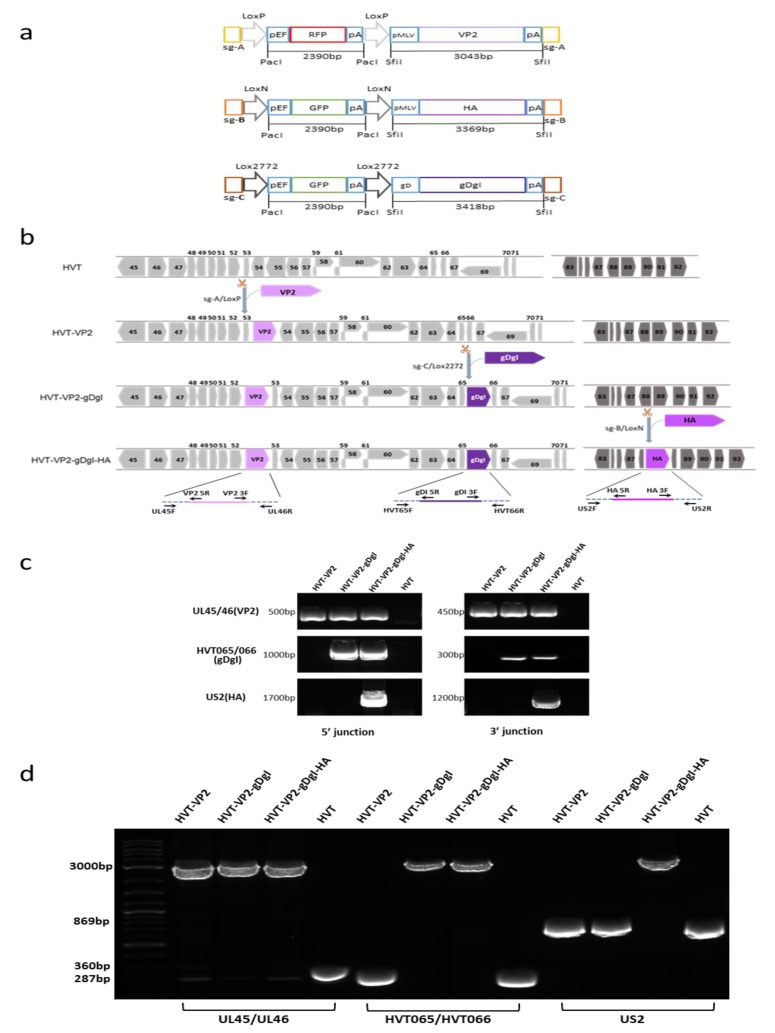
Construction of triple insert recombinant HVT-VP2-gDgI-HA. (**a**) A schematic representation of the cloning strategy for construction of three donor plasmids. The key elements include Cas9 target sites (sgA, sgB, or sgC) for releasing the insert, Lox sequences flanking the RFP/GFP cassette (LoxP, LoxN, or Lox2272) for the excision of the reporter, and the foreign gene expression cassette (VP2, HA, or gDgI) for insertion. (**b**) Herpesvirus of turkeys (HVT) genome regions spanning the three insertion sites used in this study with numbered HVT genes in grey/dark grey arrows and foreign gene expression cassettes VP2 (light purple, GenBank accession number: D00869), HA (purple, GenBank accession number MT039488.1) and gDgI (dark purple, GenBank accession number of ILTV: MN335811.1). The scissor icon represents the gRNA targeting sites in the HVT genome for foreign gene insertion. The name and position of the primers used for each PCR are shown in arrows at the bottom. (**c**) 5′ and 3′ junction PCR to confirm the correct insertion of genes in the recombinant HVT virus, using primers shown in Figure 1b. The specific bands indicate that insertions were present in the recombinant viruses. The sizes of the fragments are indicated on the left. (**d**) PCR analysis of the inserted foreign gene cassettes using primers at the flanking regions of the insertion sites. While lower bands indicate the PCR product without foreign gene insertion, the upper bands (approx. 3000 bp) show the presence of foreign gene cassettes in recombinant viruses. The sizes of the fragments are indicated on the left.

The detailed procedures for generation of recombinant HVT are described previously [23]. For the generation of HVT-VP2-gDgI, donor plasmid pGEM-sgC-Lox2272-GFP-gDgI and Cas9/gRNA expression plasmids targeting both HVT65/66 region of HVT-VP2 and the donor plasmid [23] were co-transfected into CEF followed by HVT-VP2 virus infection. GFP fluorescent single cells were isolated by fluorescence activated cell sorting, as described previously [25]. After excision of the GFP expression cassette by Cre treatment, the purified virus was identified by PCR using primers shown in Figure 1b. The resulting recombinant HVT-VP2-gDgI was then used for insertion of HA expression cassette following the same procedure generating HVT-VP2-gDgI–HA. 

### 2.4. Western Blot Analysis

1.5 × 10^5^ primary CEF were seeded into 24-well plates the day before infection. The following day, the titrated virus was recovered from the liquid nitrogen and thawed quickly in 37 °C water bath. After mixing the content of the vial, the virus was diluted to 1.5 × 10^4^ pfu/mL. One hundred microliters making final dose of 0.01 pfu/cell was added to each well of 24-well plates with growth medium and the cells were then incubated at 38.5 °C and 5% CO_2_. 48 h post infection, the infected CEF cells were collected and boiled with TruPAGE LDS sample buffer (Sigma) for 10 min. The samples were separated on a 4% to 12% TruPAGE precast gel, and the resolved proteins were transferred onto polyvinylidene difluoride (PVDF) membranes. Expression of VP2, gDgI, HA and vNr-13 were detected using anti-VP2 monoclonal antibody (MAb) (Clone ID: EU0205 CAEU Company, Beijing, China, which specifically recognizes amino acids 394-410 of VP2), anti-ILTV chicken serum (Charles River Laboratories, 10100470), anti HVT-encoded Bcl-2 homologue vNr-13 (MAb) (clone EG2 generated at Pirbright Institute) and anti-H9N2 chicken serum (generated at Binzhou Animal Science and Veterinary Medicine Academy), respectively. After probing with primary antibodies, the blots were incubated with secondary antibody IRDye^®^680RD (LI-COR Biosciences, Lincoln, NE, USA) goat anti-mouse IgG (Li-Cor) (for detection of VP2 and vNr-13), IRDye^®^800CW Donkey anti-Chicken IgG (Li-Cor) (for detection of gDgI and HA), and the results were visualized using Odyssey Clx (Li-Cor).

### 2.5. Indirect Immunofluorescence Analysis (IFA)

**Figure 2:**
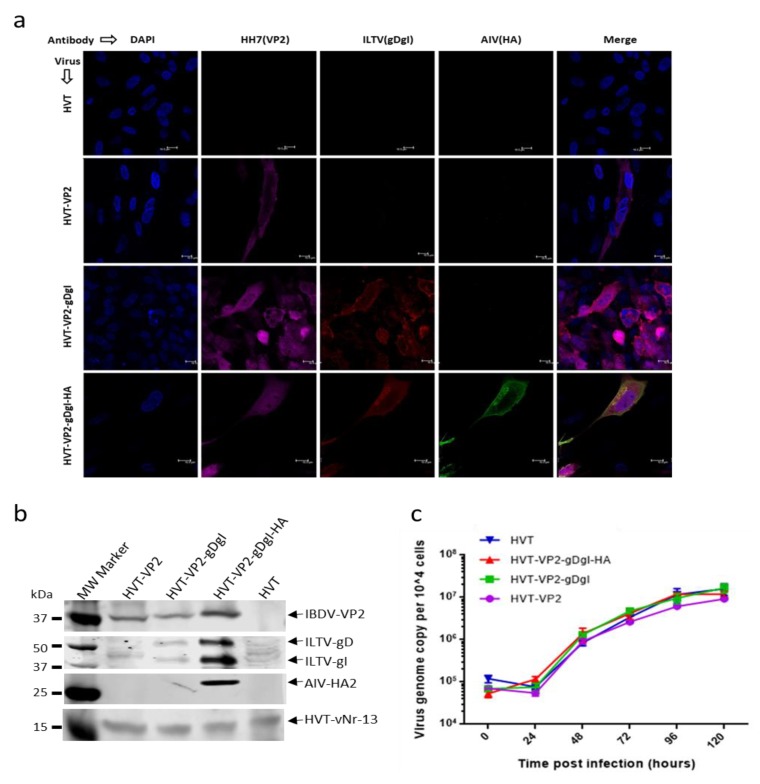
Characterization of the recombinant HVT viruses. (**a**) Detection of VP2, glycoprotein D-glycoprotein I (gDgI), and HA expression from the recombinant viruses in CEFs with IFA. VP2 expression was detected with MAb HH7 followed by goat anti-mouse IgG labelled with Alexa Fluor 633 (Far red). gDgI expression was detected with anti-ILTV chicken serum followed by goat anti-chicken IgG labelled with Alexa Fluor 568 (red). HA expression was detected with anti-H9N2 chicken serum followed by goat anti-chicken IgG labelled with Alexa Fluor 488 (green). The nuclei were stained with DAPI (blue). (**b**) Detection of VP2, gDgI and HA expression in recombinant virus infected CEF by Western blotting with the same primary antibodies described in (A). HVT-encoded Bcl-2 homolog vNr-13 was used to confirm HVT infection. (**c**) The growth kinetics of recombinant HVT viruses. In vitro growth rates of HVT-WT, HVT-VP2, HVT-VP2-gDgI and HVT-VP2-gDgI-HA measured from the viral genome copy numbers determined using TaqMan real-time qPCR on DNA extracted from chick embryo fibroblasts (CEF) harvested at various time points after inoculation. Viral genome copy numbers per 10,000 cells (shown with 95% confidence intervals) are shown on the y axis.

**Figure 3:**
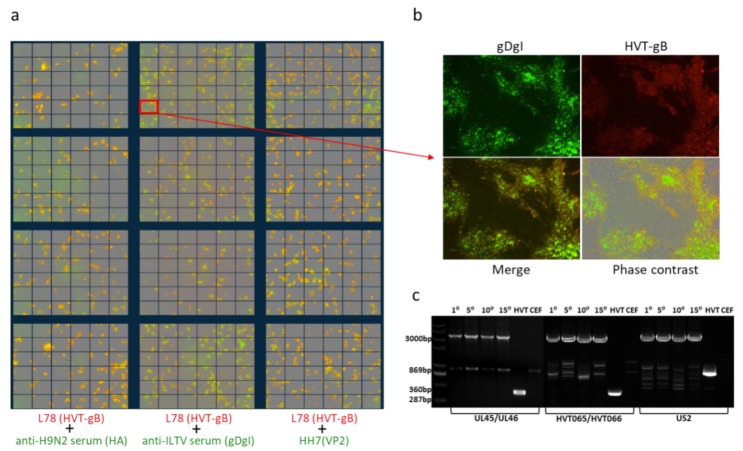
Stability of triple insert recombinant HVT-VP2-gDgI-HA. (**a**) Merged images of double stained HVT-VP2-gDgI-HA infected CEF with anti-HVT-gB MAb antibody L78 (red) and anti-H9N2 chicken serum (green) (left panel), anti-ILTV chicken serum (green) (middle panel) or anti-VP2 MAb HH7 (green) (right panel). Images were taken from 36 separate regions per well and 4 wells per sample in 24-well plates using the IncuCyte. (**b**) The marked region of the merged images of gDgI and HVT-gB staining in (a) is shown enlarged with gDgI staining (green, top left), HVT-gB staining (top-right), the merged image under UV light (bottom left) and phase contrast (bottom right). (**c**) PCR to confirm the presence/absence of the VP2 expression cassette at UL45/46 with primers UL45F/UL46R, gDgI at HVT65/66 with primers HVT65F/HVT66R and HA at US2 region with primers US2F/US2R from the recombinant virus HVT-VP2-gDgI-HA at passage 1, 5, 10, and 15 in CEFs (primer sequence shown in Figure 2b). HVT infected and noninfected CEF were included as controls. The absence of a lower band, during amplification, in the recombinant virus during passage indicates that the recombinant virus is stable. The sizes of the fragments are indicated on the left.

The same dose and infection procedure described above was followed. Forty-eight hours post infection, CEF cells in 24 well plates (and on coverslips—Figure 2 only) were fixed with 4% chilled paraformaldehyde and permeabilized with 0.1% Triton X-100 in phosphate buffer. In Figure 2a, the cells were labelled with: MAb HH7 (generated at Pirbright Institute) followed by goat anti-mouse IgG labelled with Alexa Fluor 633 (Invitrogen) to detect VP2 expression, Anti-ILTV chicken serum followed by goat anti-chicken IgG labelled with Alexa Fluor 568 to detect gDgI expression, and Anti-H9N2 chicken serum (generated at Binzhou Animal Science and Veterinary Medicine Academy) followed by goat anti-chicken IgG labelled with Alexa Fluor 488 (Invitrogen) were to detect HA expression. Cell nuclei were then stained with 4′, 6-diamidino-2-phenylindole (DAPI), coverslips were mounted in Vectashield onto microscope slides and imaged in a Leica TCS SP5 confocal laser scanning microscope (Leica Microsystems, Milton Keynes, UK). In Figure 3a, the cells were labelled with: MAb L78 (generated at Pirbright Institute) followed by goat anti-mouse IgG labelled with Alexa Fluor 568 to detect HVT-gB expression, HH7 followed by goat anti-mouse IgG labelled with Alexa Fluor 488 (Invitrogen) to detect VP2 expression, anti-ILTV chicken serum followed by goat anti-chicken IgG labelled with Alexa Fluor 488 to detect gDgI expression, and anti-H9N2 chicken serum followed by goat anti-chicken IgG labelled with Alexa Fluor 488 to detect HA expression. An IncuCyte was used to image 36 separate regions per well and 4 wells per sample.

### 2.6. Stability of the Inserted Genes in the Recombinant Viruses

The recombinant viruses HVT-VP2, HVT-VP2-gDgI and HVT-VP2-gDgI-HA were passaged through CEF cells 15 times. The stability of each inserted cassette was monitored by PCR with primer pairs located at the flanking region of the insertion site (UL45F and UL46R for VP2 expression, HVT65F and HVT66R for gDgI expression cassette, US2F and US2R for HA expression cassette) using DNA extracted from every 5th passage. The primer sequences used for PCR described above are listed in Table 3.

### 2.7. In Vitro Growth Kinetics

To investigate the growth properties of the recombinant HVT, CEF cultured in 6-well plates were infected with 100 pfu of each virus per well and harvested at 0, 12, 24, 48, 72, 96, and 120 h post infection. DNA was extracted using the DNeasy 96 Blood & Tissue kit (Qiagen, Germantown, IL, USA) and used for real-time q-PCR to determine the in vitro growth kinetics of the viruses using methods described previously [2]. Duplex real-time q-PCR to detect both the HVT SORF1 gene and chicken ovotransferrin gene enabled calculation of HVT genome copies per 10,000 cells using a dilution series of pHVT BAC3 DNA [2] and p-GEM-T-ovo [29] to produce standard curves. The HVT genome copies per 10,000 cells were plotted against hours post-infection for each of the viruses. 

## 3. Results

### 3.1. Generation of Triple Insert HVT Recombinant HVT-VP2-gDgI-HA

To generate triple insert HVT recombinant, we inserted ILTV gDgI and AIV H9HA expression cassettes sequentially into the HVT-VP2 virus, reported previously [23], to generate HVT-VP2-gDgI-HA. In order to distinguish between the different donor templates and to ensure that the reporter cassette was correctly excised at each stage, different bait sequences (sgB target sites for HA and sgC target sites for gDgI) were introduced at the ends of the donor templates for releasing the donor sequences. Different variant Lox sequences (LoxN for HA and Lox2272 for gDgI) facilitated marker gene excision by Cre recombinase (Figure 1a). Insertion of the gDgI and HA expression cassettes into the HVT-VP2 genome at HVT65/66 and US2 loci respectively were carried out sequentially using the strategy described previously [23]. After each insertion and reporter excision, we examined the correct location and orientation of the insert by junction PCR (5′ and 3′) and the purity of the purified plaque was determined by PCR using primers outside of the insertion sites (Figure 1b). Wild type HVT-infected cells were used as negative control. As expected, junction PCR of both 5′ and 3′ for VP2 insertion between UL45/46 was amplified for all three recombinants HVT-VP2, HVT-VP2-gDgI, and HVT-VP2-gDgI-HA; junction PCR of both 5′ and 3′ for gDgI insertion between HVT-65/66 was amplified for two recombinants HVT-VP2-gDgI and HVT-VP2-gDgI-HA; junction PCR of both 5′ and 3′ for HA insertion at US2 region was amplified only for recombinant HVT-VP2-gDgI-HA when HA was inserted (Figure 1c). In contrast, no PCR product was amplified for wild type HVT at any insertion site. Similarly, when the outside primers were used, the full-length inserts with the flanking sequences of VP2 were present in all three HVT recombinant viruses. Insert of gDgI was present in the two recombinants HVT-VP2-gDgI and HVT-VP2-gDgI-HA. HA was only present in HVT-VP2- gDgI-HA virus. The smaller band without insert was present only in wild type HVT and the recombinants before the corresponding donor were inserted, indicating the purified virus only contained the recombinant HVT with correct insert/inserts (Figure 1d). All of the data above confirmed that gDgI and HA were correctly inserted in the purified recombinant virus stocks.

### 3.2. Characterization of Recombinant HVT-VP2-gDgI and HVT-VP2-gDgI-HA

We next investigated the expression of IBDV VP2, ILTV gDgI, and AIV HA by immunofluorescence labelling of different recombinant virus infected CEF cells with monoclonal antibody HH7 (VP2 specific), ILTV-specific chicken serum (for gD and gI detection) and H9N2-infected chicken serum (for HA detection). HVT-infected cells were used as a negative control. As shown in Figure 2a, VP2 expression (Far-red) was observed in all three recombinant virus-infected CEFs but not HVT-infected cells. Expression of gDgI (red) was detected in HVT-VP2-gDgI and HVT-VP2-gDgI-HA infected cells, whereas HA (green) was only expressed in CEF infected with HVT-VP2-gDgI-HA. Results indicate that the three inserts were expressed correctly from each of the constructs. 

For further assessment of the expression of each protein, cell lysates from CEF infected with each recombinant virus were analyzed by Western blotting (Figure 2b). The HVT-vNr-13-specific MAb EG2 was used as an infection/loading control. Again, IBDV VP2-positive band was observed in all three recombinant virus infections, while bands representing gD and gI expression were present in both HVT-VP2-gDgI and HVT-VP2-gDgI-HA infected cells although the non-specific bands are present in HVT-VP2 and HVT infected cells. HA expression was only observed in HVT-VP2-gDgI-HA infected CEF, further confirming the correct expression of each insert by the recombinant viruses in the infected CEF. 

Having confirmed the presence and expression of all three inserts, we examined whether the insertion of these additional expression cassettes influenced the growth of the recombinant viruses. Primary CEFs, grown in 6-well plates, were infected with 100 pfu of either HVT or HVT recombinants with one, two or three inserts. The viral genome copy numbers per 10,000 cells at various days post-infection were examined by qPCR to determine the virus replication rates. As shown in Figure 2c, the growth curves were similar between the wild type and all the recombinant HVTs.

### 3.3. Stability of Recombinant HVT-VP2-gDgI-HA

Previously we have shown that the VP2 expression cassette was stably integrated into the HVT genome during repeated cell passages [23]. To determine the stability of the newly inserted two expression cassettes during continuous passage of triple insert recombinant virus, HVT-VP2-gDgI-HA was sequentially passaged on primary CEFs and infected cells at the 15th passage were examined by fluorescence imaging. The expression of each insert was examined, together with MAb anti-HVT-gB L78 for HVT infection control, to identify the double stained cells demonstrating the stable integration of the foreign gene. The loss of the insert from any of the recombinant viruses during the passage would be apparent, as these cells would only show positive labelling with the anti-HVT-gB antibody. As shown in Figure 3a,b, the presence of dual labelling in all of the infected cells demonstrated that all of the three inserts were stably integrated into the HVT genome.

To further confirm that the three inserts were indeed stably integrated during the repeated sequential passages, viral DNA was extracted and analyzed after every 5 passages by PCR using outside primers, as described above. As shown in Figure 3c, the small bands amplified from HVT infection are the predicated size for the sequence spanning the gRNA target site before insertion, the large band in the recombinant virus-infected cells are the predicted size for the sequence containing the insert. Although some of the non-specific bands are present, the correct sized small and large specific bands are clearly distinguishable. The absence of the smaller bands in recombinant virus-infected cells after repeated cell culture passages indicated that both gDgI and HA expression cassettes were stably integrated into the HVT-VP2 genome. The stability of previously inserted VP2 was not affected following the insertion of the two additional foreign genes. 

## 4. Discussion

With the requirement of up to 15 different vaccines to protect against multiple avian diseases, there is great demand for developing multivalent recombinant vaccines that can induce simultaneous protection against multiple avian pathogens. Among the recombinant viral vectors, HVT is the most successful and widely used commercially for the delivery of various immunogenic proteins to protect against poultry diseases such as NDV, IBDV, and AIV. With the additional value of their use as a hatchery vaccine, recombinant HVT vaccines are effective against the specific antigens expressed. However, when different recombinant vaccines are combined to obtain protection against multiple avian pathogens, the protection levels are less than optimal to each of the components, due to the interference between recombinant HVTs expressing different antigens. Although the precise mechanisms of such interference are not fully clear, this limitation restricts the combined use of the different recombinant HVT vectors. The recent successful use of CRISPR/Cas9 gene editing for rapid integration of a foreign gene into the HVT genome [23] has promoted us to insert more foreign genes for multivalent HVT vaccine development using the same strategy. 

A previous report showed that the insertion site has a major influence in the successful expression of foreign gene [30]. Several genomic loci in the HVT genome, including US2, US10, intergenic regions of UL45/UL46 and HVT065/HVT066, have been shown to be suitable for foreign gene insertion. Following the successful insertion of the VP2 expression cassette into HVT at UL45/UL46 region, we chose to insert the gDgI expression cassette into the HVT065/HVT066 intergenic region and the H9 expression cassette into the US2 gene using the same strategy. Indeed, both expression cassettes were successfully integrated into the desired location and remained stable after 15 passages, as confirmed by both immunostaining and PCR. Furthermore, the fact that the recombinant viruses with one, two and three foreign gene insertions had similar growth to the wide type virus has further confirmed the suitability of these sites for foreign gene insertion. 

Apart from the features of an excisable fluorescence selection marker for easy visualization of recombinant virus and the unique SfiI sites for swapping the foreign gene expression cassette of interest, new features including two new universal gRNA target sequences for releasing the donor template and two more Lox variant sequences to avoid repeated usage of the LoxP sequence for marker excision of the first insert have been introduced for donor vector construction. This has made different donor templates easily distinguishable. The only limitation for the whole procedure is the complexity of the identification of GFP/RFP-positive clones by junction PCR as the inserted sequence could be in either orientation. The primers for junction PCR described here is only for identification of the recombinant inserted in the sense orientation. The two primers located at each end of the inserted sequence could be swapped to identify the insert in antisense orientation. In the current study, the foreign gene expression cassettes were inserted sequentially, however, the insertion of different donor templates could be done simultaneously with the approach of using donor templates with different colored fluorescence marker genes. Similarly, subsequent marker gene excision can also be done simultaneously with different Lox variant sequences, making the whole process more rapid and efficient. The same approach can also be used for the development of new multivalent vectored vaccines on backbone of serotype 1 and 2 of MDV vaccine strains such as CVI988 and SB-1, other herpesvirus such as ILTV and DEV as well as other avian DNA viruses including adenoviruses and pox viruses. With prevention being the most effective means of controlling virus infection, protecting against multiple diseases with fewer injections of a multivalent vectored vaccine improves poultry welfare by reducing the stress associated with multiple needle sticks, speeds the vaccination processes, reduces the cost to produce and holds great promise for poultry industry. 

## Figures and Tables

**Table 1:** sgRNA targeting sequences in HVT * genome and donor constructs.

sgRNA	Target Sequences	PAM	Gene Locus
UL45/46	GAGATCGAGTGCCGCATCAC	CGG	Between UL45 & UL46
HVT65/66	GGGAAACTAAATGTTCATAG	AGG	Between HVT65 and HVT66
US2	ACACAAATTGCGTTTAGGTG	GGG	US2
sg-A	GAGATCGAGTGCCGCATCAC	CGG	sgA
sg-B	GAGATCGAGTTCGGCTAGAC	CGG	sgB
sg-C	GAGAGAGTGTGGCGACTCTG	CGG	sgC

* Genebank accession number: NC_002641.

**Table 2:** Primer sequences used for donor construction.

Primer	Sequences
SfiIx2-F	CTAGCAAGGCCGCCTAGGCCGGCGCGCCGTTAAACGGCCATTATGGCCGTTT
SfiIx2-R	AAACGGCCATAATGGCCGTTTAACGGCGCGCCGGCCTAGGCGGCCTTG
sgB-LoxN-F	CATGGGAAGTCGAGTTCGGCTAGACCGGATAACTTCGTATAAGGTATACTATACGAAGTTATTTAATTAAATAACTTCGTATAAGGTATACTATACGAAGTTATGGCCGCCTAGGCCGGCGCGCCGTTTAAACGGCCATTATGGCCGAAGTCGAGTTCGGCTAGACCGGCA
sgB-LoxN-R	TATGCCGGTCTAGCCGAACTCGACTTCGGCCATAATGGCCGTTTAAACGGCGCGCCGGCCTAGGCGGCCATAACTTCGTATAGTATACCTTATACGAAGTTATTTAATTAAATAACTTCGTATAGTATACCTTATACGAAGTTATCCGGTCTAGCCGAACTCGACTTCC
sgC-Lox2272-F	CATGGGAGAGAGTGTGGCGACTCTGCGGATAACTTCGTATAAAGTATCCTATACGAAGTTATTTAATTAAATAACTTCGTATAAAGTATCCTATACGAAGTTATGGCCGCCTAGGCCGGCGCGCCGTTTAAACGGCCATTATGGCCGAGAGAGTGTGGCGACTCTGCGGCA
sgC-Lox2272-R	TATGCCGCAGAGTCGCCACACTCTCTCGGCCATAATGGCCGTTTAAACGGCGCGCCGGCCTAGGCGGCCATAACTTCGTATAGGATACTTTATACGAAGTTATTTAATTAAATAACTTCGTATAGGATACTTTATACGAAGTTATCCGCAGAGTCGCCACACTCTCTCC
HA-F *	CGAGCGGCCGCATGGAAGCACTATCACTGATAACTATAC
HA-R	GCGGCGGCCGCTTATATACAAATGTTGCATCTGCAAGA

* HA sequence with GenBank accession number MT039488.1 is from Shandong isolate A/chicken/China/DZL018/2016(H9N2).

**Table 3:** Primers used for junction PCR and knock-in detection.

Primer	Sequence
UL45F	TACCGTTATATGTCAGCGACCCA
UL46R	CTCCGACAACCAAATACTTTCATGA
HVT65 F	TCGCTATGCAAAGAGATGCGTG
HVT66 R	CGTCTGCGATAACTACGCCT
US2F	CTGTGATACACTTGGGAGCC
US2R	GACGTTTCCGATCTTCCACA
VP2 5R	GTGCATGACCGTGCTGATTC
VP2 3F	CGTCTTGGCATCAAGACCGT
gDI 5R	ACGACAGGCACATTAGCTGGACC
gDI 3F	TAGTTACTGTGCCTTCTAGTTGCCAG
HA 5R	GCTCTGTGTGGAGCAGTTCT
HA 3F	TGAAGAGAGCGTTGGGTTCC
